# Novel male-biased expression in paralogs of the aphid *slimfast *nutrient amino acid transporter expansion

**DOI:** 10.1186/1471-2148-11-253

**Published:** 2011-09-14

**Authors:** Rebecca P Duncan, Lubov Nathanson, Alex CC Wilson

**Affiliations:** 1 Department of Biology, University of Miami, Coral Gables, FL 33146, USA; 2 J.P.Hussman Institute for Human Genomics, Miller School of Medicine, University of Miami, Miami, FL 33136, USA

## Abstract

**Background:**

A major goal of molecular evolutionary biology is to understand the fate and consequences of duplicated genes. In this context, aphids are intriguing because the newly sequenced pea aphid genome harbors an extraordinary number of lineage-specific gene duplications relative to other insect genomes. Though many of their duplicated genes may be involved in their complex life cycle, duplications in nutrient amino acid transporters appear to be associated rather with their essential amino acid poor diet and the intracellular symbiosis aphids rely on to compensate for dietary deficits. Past work has shown that some duplicated amino acid transporters are highly expressed in the specialized cells housing the symbionts, including a paralog of an aphid-specific expansion homologous to the *Drosophila *gene *slimfast*. Previous data provide evidence that these bacteriocyte-expressed transporters mediate amino acid exchange between aphids and their symbionts.

**Results:**

We report that some nutrient amino acid transporters show male-biased expression. Male-biased expression characterizes three paralogs in the aphid-specific *slimfast *expansion, and the male-biased expression is conserved across two aphid species for at least two paralogs. One of the male-biased paralogs has additionally experienced an accelerated rate of non-synonymous substitutions.

**Conclusions:**

This is the first study to document male-biased *slimfast *expression. Our data suggest that the male-biased aphid *slimfast *paralogs diverged from their ancestral function to fill a functional role in males. Furthermore, our results provide evidence that members of the *slimfast *expansion are maintained in the aphid genome not only for the previously hypothesized role in mediating amino acid exchange between the symbiotic partners, but also for sex-specific roles.

## Background

A fundamental goal of molecular evolutionary biology is to understand the evolutionary fate and biological consequences of duplicated genes. Most duplicate genes fail to reach fixation [1], strongly suggesting that retained gene duplicates provide a selective advantage. Recent genome sequencing projects have revealed that the water flea, *Daphnia pulex*, and the pea aphid, *Acyrthosiphon pisum*, are unique among arthropods in having exceptionally high levels of tandem, lineage-specific gene duplication and retention [2-9]. Apart from high gene duplication levels, both *Daphnia *and aphids, in contrast to other sequenced insects, are cyclical parthenogens with complex life cycles characterized by extensive polyphensim [2,3]. The shared life cycle complexity and gene duplication levels of aphids and *Daphnia *suggest that polyphenism may be a major factor underlying the extensive gene duplications characterizing these two genomes.

Several aphid-specific gene family expansions have been suggested to be associated with the aphid life cycle and polyphenism [3]. For example, several genes involved in early developmental signaling pathways have undergone aphid-specific duplications [4], which may account for the differences observed in embryonic development between live-bearing, asexual females and egg-laying, sexual females [4,10]. One major developmental difference between asexual and sexual females is the type of cell division that initiates oogenesis. While sexual female oocytes undergo standard meiosis, asexual female oocytes undergo a modified form of meiosis before initiating development of a parthenogenetic embryo [11]. The differences in cell division between asexual and sexual female oocytes might be accommodated by gene duplications found in cell cycle genes, some of which are differentially expressed in sexual and asexual female ovarioles [11]. Additionally, since different morphs within the same clonal line are genetically identical, their morphological, biological and ecological differences must result from differences in gene expression [e.g. [12]], which may be facilitated by duplicate, divergent chromatin remodeling genes [6].

In contrast to an hypothesized association with polyphenism, some aphid-specific gene duplications appear to be more connected with other aspects of aphid biology, such as their diet. Aphids feed on plant phloem sap, a diet rich in sugar, which creates a high positive osmotic potential between the ingested phloem sap and the aphid hemolymph. This osmotic challenge must be overcome in order for aphids to avoid desiccation [8,13]. Aphids cope with their osmotically challenging diet both by voiding excess sugar as liquid waste called honeydew [14], and by transporting sugars across the gut epithelium into their hemolymph via sugar transporter proteins [8]. Interestingly, aphid sugar transporters have undergone aphid-specific gene duplications [8], which may enable aphids to utilize a sugar rich diet.

Another group of diet-associated genes that have undergone aphid-specific duplications are the nutrient amino acid transporters [9]. Duplications in nutrient amino acid transporters may be explained by another feature of the aphid phloem sap diet. Apart from being rich in sugar, phloem sap is also deficient in essential amino acids. Essential amino acids cannot be synthesized *de novo *by metazoans, so their deficit in phloem is compensated for by the intracellular bacterial symbiont, *Buchnera aphidicola *[15,16]. *Buchnera *reside within membrane-bound compartments in specialized aphid cells called bacteriocytes, where they exchange non-essential amino acids from the aphid for the essential amino acids they synthesize [reviewed in [16]]. Recently, we discovered that some nutrient amino acid transporters are highly expressed in bacteriocyte cells where *Buchnera *reside, suggesting that these amino acid transporters mediate amino acid exchange between aphids and *Buchnera *[9]. Our finding that bacteriocyte-enriched amino acid transporters are often members of gene family expansions suggests that past duplication events enabled some duplicates to conserve their ancestral function while others diverged to fill a role in symbiosis [9].

Here we propose that in addition to a role in symbiosis, duplicate nutrient amino acid transporters have evolved sex-biased roles. In microarray data for the aphid species *Myzus persicae*, we unexpectedly discovered that two nutrient amino acid transporters have highly male-biased expression. These transporters, orthologous to the *Drosophila *gene *slimfast*, were nested within an aphid-specific *slimfast *expansion among 10 *A. pisum *paralogs. Male-biased expression for these two paralogs is conserved in *A. pisum *and detailed expression analysis in *A. pisum *revealed one additional male-biased *slimfast *paralog and one asexual female-biased *slimfast *paralog. Together, these data suggest that, in addition to the symbiosis-based functions proposed by Price *et al.*, [9], the aphid-specific *slimfast *expansion is maintained for sex-biased functions.

## Results

### Microarray and automated annotation results

We mined a microarray comparing expression between male, sexual female, and asexual female *Myzus persicae *aphids for genes with differential expression between males and at least one female morph (*i.e*. genes that were not sex-neutral). Comparisons were made with two types of asexual females: asexual females from perpetually parthenogenetic cultures requiring higher temperatures and long day lengths (long day asexual females) and asexual females collected from sexual cultures, induced by lower temperatures and shortened day lengths (short day asexual females). Comparing males and sexual females to both long and short day asexual females enabled us to control for day length effects in gene expression (since sexual morphs are only present under short day conditions).

Genes with differential expression between males and at least one female reproductive morph are referred to as "male-enriched" or "female-enriched". These sex-enriched genes were identified by fold change (4-fold or greater upregulation) and significance (False discovery rate (FDR) < 0.05). Of the 10,478 *M. persicae *unigenes represented on the microarray, 768 (~7%) were male-enriched and 725 (~7%) were female-enriched (Additional file 1; a list of all sex-enriched genes with descriptions, annotations and fold changes can be found in Additional files 2 and 3). Of the male-enriched genes, 126 (16%) were overexpressed in males relative to all three female morphs and of the female-enriched genes, 82 (11%) were enriched in all three female morphs relative to males. We refer to genes that are differentially expressed between males and all three female reproductive morphs as "male-biased" or "female-biased". One gene was enriched in both sexes depending upon the female morph: contig 2728 (see Additional file 4 for corresponding EST accession numbers) was overexpressed in males relative to sexual females and overexpressed in asexual females relative to males (Additional files 1, 2, and 3).

Mapping and annotation results obtained from Blast2GO are summarized in Additional file 1. The homology search returned significant BLAST hits for 501 (65%) male-enriched and 554 (76%) female-enriched genes. Gene Ontology (GO) terms were found for 371 (48%) male-enriched and 414 (57%) female-enriched genes. Round one annotation, which excluded electronic annotations, annotated 202 (26%) male-enriched and 291 (40%) female-enriched genes. Round two annotation, which included electronic annotations, annotated an additional 63 (8%) male-enriched and 51 (7%) female-enriched genes. InterProScan [17] added annotations to genes annotated in the previous steps and additionally annotated 42 (5%) male-enriched and 31 (4%) female-enriched genes that were previously unannotated. Annotations were assigned to a total of 307 (40%) male-enriched and 373 (51%) female-enriched genes (Additional file 1).

Overrepresented GO terms are presented in Additional file 5. In total, 75 GO terms were significantly overrepresented in sex-enriched genes (FDR < 0.05). Of these GO terms, 16 (21%) were overrepresented in males and 59 (79%) in females. Female-enriched genes were represented by several GO classes such as nucleotide binding, nitrogen metabolism, cell cycle, cytoskeleton organization and organelles. In contrast, male-enriched genes were dominated by GO terms related to transporter and channel activity (Additional file 5).

### Identification and annotation of sex-enriched nutrient amino acid transporters in the aphid *Myzus persicae*

From the sex-enriched genes identified in the microarray, we identified five nutrient amino acid transporters overexpressed 4.2-fold to 20.1-fold in males (FDR < 0.05; Table 1 and Additional file 3). Four of the five male-enriched transporters were annotated in Blast2GO based on GO terms associated with significant BLAST hits (E < 0.001) and/or InterProScan IDs (Table 1 Additional file 3). The fifth transporter had no BLAST hits associated with GO terms, but its one InterProScan ID was annotated as belonging to transmembrane amino acid transporters (Table 1). After translating unigene nucleotide sequences to amino acid sequences based on the largest open reading frame, four of the five transporters significantly matched Pfam domains *AA_permease *(PF000324) or *Aa_trans *(PF01490) (Table 1).

**Table 1:** *Myzus persicae *male-enriched nutrient amino acid transporters identified in microarray and annotations

Contig ID^a^	GO terms	InterProScan IDs	Pfam domain	Fold change (Compared to)^b^
1492	GO:0006629: lipid metabolic process GO:0005215: transporter activity GO:0007165: signal transduction GO:0005886: plasma membrane GO:0006810: transport GO:0040007: growth GO:0006811: ion transport	IPR002293: Amino acid/polyamine transporter IPR004841: Amino acid permease domain IPR015606: Cationic amino acid transporter	AA_permease (PF00324)	9.5-15.0 (AFL) 6.9-10.5 (AFS) 13.4-20.1 (SF)
3389	GO:0006810: transport	IPR013057: Amino acid transporter, transmembrane	Aa_trans (PF01490)	4.2 (AFS)
4712	Not annotated in Blast2GO	IPR013057: Amino acid transporter, transmembrane	Aa_trans (PF01490)	5.5 (SF)
4891	GO:0005623: Cell	IPR013057: Amino acid transporter, transmembrane	Aa_trans (PF01490)	9.3 (SF)
8321	GO:0009605: response to external stimulus GO:0006950: response to stress GO:0006629: lipid metabolic process GO:0006810: transport GO:0050789: regulation of biol. process GO:0005634: nucleus GO:0005623: Cell GO:0005215: transporter activity GO:0040007: growth GO:0006139: nucleobase, nucleoside, and nucleic acid metabolic nucleotide proc. GO:0009058: biosynthetic process GO:0005886: plasma membrane GO:0006811: ion transport	IPR002293: Amino acid/polyamine transporter IPR015606: Cationic amino acid transporter	No significant Pfam hits	5.7 (AFL) 7.2 (AFS) 7.6 (SF)

^a ^Unigenes targeted in microarray were based on contigs assembled from ESTs generated in [40]. Contigs are available for download at http://www.aphidbase.com/aphidbase/downloads. See Additional file 4 for GenBank accession numbers corresponding to each contig.

^b^AFL: Asexual female at long day; AFS: Asexual female at short day; SF: Sexual female

**Figure 1:**
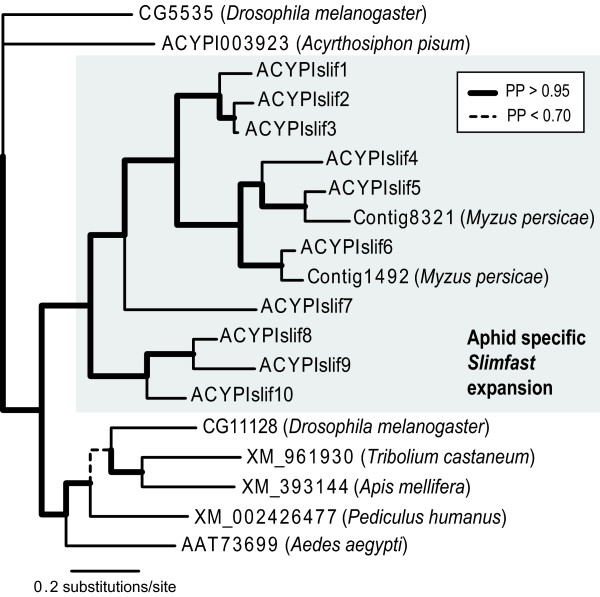
**Phylogeny of the *slimfast *gene family**. Phylogeny was reconstructed in MrBayes v. 3.1.2. The aphid specific *slimfast *expansion is shown in the gray box. Posterior probability support for relationships is represented by branch width and style, as indicated in the key. Outgroup sequences were shown by Price *et al.*, [9] to belong to a closely related clade of the *slimfast *gene family. Gene IDs beginning with "*ACYPI*" belong to *A. pisum *and gene IDs beginning with "contig" belong to *M. persicae*.

Two transporters (contigs 1492 and 8321; See Additional file 4 for the GenBank accession numbers) were overexpressed 5.7 to 20.1-fold in males relative to both sexual and asexual females (Table 1 Additional file 3). We further annotated these two transporters based on their strongly male-biased expression. Using BLASTX and reciprocal TBLASTN searches, *M. persicae *contig 1492 (Table 1 Additional file 4) and the *A. pisum *amino acid permease ACYPI005156 were reciprocal BLAST best hits. These two genes also paired with unambiguous support in a gene phylogeny for insect orthologs to the *Drosophilia *gene *slimfast*, a cationic amino acid permease [18] of which *A. pisum *has a ten paralog aphid-specific expansion [9] (Figure 1). A BLASTX search for the other *M. persicae *contig, 8321 (Table 1 and Additional file 4), against the *A. pisum *nr protein database returned the *A. pisum *gene ACYPI003240 as the best hit, but the reciprocal TBLASTN search failed to return contig 8321 as the best hit for ACYPI003240. Phylogenetic analysis paired contig 8321 and ACYPI003240 with unambiguous support in the *slimfast *gene phylogeny (Figure 1). Based on the homology of the aphid expansion to *slimfast*, we named the 10 *A. pisum *paralogs *ACYPIslif1-10 *(Table 2).

**Table 2:** *Acyrthosiphon pisum slimfast *gene identifications

*A. pisum slimfast *name	*A. pisum *gene ID^a^
*ACYPIslif1*	ACYPI000537
*ACYPIslif2*	ACYPI000584
*ACYPIslif3*	ACYPI005472
*ACYPIslif4*	ACYPI003523
*ACYPIslif5*	ACYPI003240
*ACYPIslif6*	ACYPI005156
*ACYPIslif7*	ACYPI005118
*ACYPIslif8*	ACYPI008904
*ACYPIslif9*	ACYPI008323
*ACYPIslif10*	ACYPI002633

^a^Gene IDs are recognized in GenBank

Intrigued by having identified male-enriched nutrient amino acid transporters, we sought to discover if more sex-enriched amino acid transporters were represented in the microarray that our annotation pipeline had failed to annotate. TBLASTX and BLASTN searches queried all 40 *A. pisum *nutrient amino acid transporters [as identified in 9] against the *M. persicae *unigenes in the microarray. Apart from the 5 male-enriched transporters annotated above, we uncovered 9 additional contigs with significant (E < 0.001) similarity to *A. pisum *nutrient amino acid transporters. Of these 9 contigs, 5 were significantly (FDR < 0.05) male-enriched (two of which were male-biased) and 3 were female-enriched. Fold change for most of these contigs was less than four, explaining why we did not identify them in our initial analysis. In total, the *M. persicae *microarray targeted 14 contigs that significantly matched nutrient amino acid transporters, of which 10 were male-enriched or male-biased and 3 were female-enriched.

### Sex-biased expression in the aphid *slimfast *expansion

In our survey for more amino acid transporters in the microarray, we found no additional *slimfast *paralogs. We thus shifted the focus of our study to *A. pisum *so as to leverage its sequenced genome [3] to analyze the evolution of male-biased aphid *slimfast*. Expression was quantified by quantitative PCR (QPCR) between males and the three female morphs across three biological replicates. Expression was quantified for all *slimfast *paralogs but three, for which sequence similarity and AT richness in the untranslated regions prevented the design of paralog-specific primers [9].

**Figure 2:**
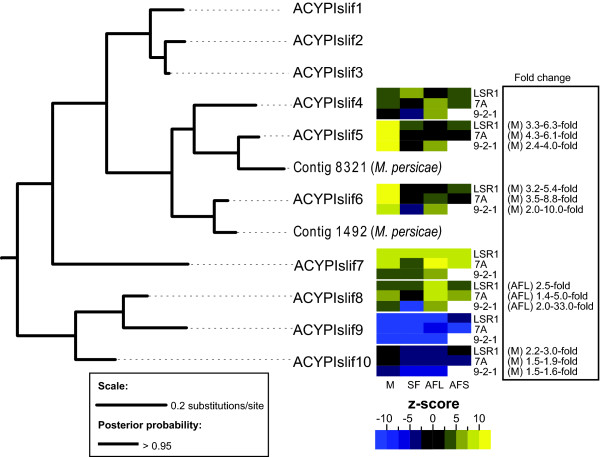
**Quantitative gene expression analysis of *Acyrthosiphon pisum slimfast *paralogs**. Gene expression profiles are shown for three *A. pisum *lineages (LSR1, 7A, 9-2-1). Transcript levels for each *slimfast *paralog were quantified by QPCR on cDNA for whole adult asexual females at long day (AFL) and short day (AFS), sexual females (SF), and males (M). The relative abundance of each paralog was normalized to the housekeeping gene *GAPDH *(ACYPI009769) and expression levels were standardized by converting them to z-scores and compiled into a heat map (for details, see methods). Expression was not quantified for *ACYPIslif1-3 *because sequence similarity and AT richness in the untranslated regions precluded our ability to design paralog-specific QPCR primers. Aphid line 9-2-1 does not produce asexual females at short day so we did not measure AFS expression for 9-2-1 (see methods for details). Yellow: z > 0; Blue: z < 0.

Expression results are presented as a heat map aligned against the *slimfast *gene phylogeny in Figure 2. Paralogs *ACYPIslif5*, *6*, and *10 *were male-biased, with *ACYPIslif5 *and *6 *showing the highest male-biased expression (Average fold change ± SD = 5.0 ± 2.2) and *ACYPIslif10 *showing less upregulation in males (Average fold change ± SD = 2.0 ± 0.5). Expression patterns for male-biased genes were consistent across all biological replicates (Figure 2). Additional patterns that were consistent across the three biological replicates were (1) enriched expression for *ACYPIslif8 *in asexual females at long day and (2) consistent low expression across all morphs for *ACYPIslif9*.

### Molecular evolution of male-biased *slimfast *paralogs

**Figure 3:**
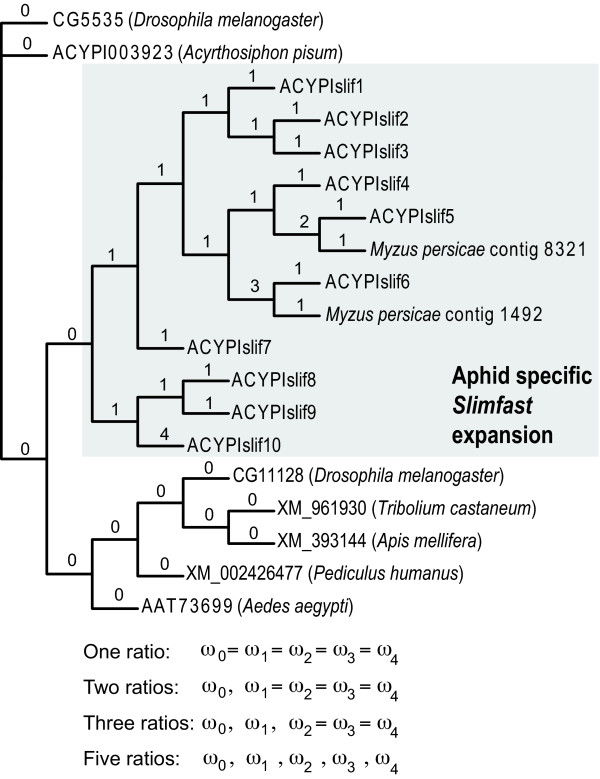
**Molecular evolution models**. Strategy used to test for accelerated rates of evolution along branches leading to male-baised aphid *slimfast *paralogs. Tree topology is identical to the phylogeny in Figure 1. Each branch was assigned a ω category as indicated. The models tested are outlined below the phylogeny. The null model (one ratio) assumed equal ω across all branches. The first alternative model (two ratios) assumed one ω ratio for branches outside the aphid-specific *slimfast *expansion and a different ω ratio for branches within the expansion. The second alternative model (three ratios) assumed an additional, collective ω ratio for the branches leading to the three male-biased paralogs, and third alternative model (five ratios) assumed a distinct ω for each branch leading to a male-biased paralog or clade.

The expression profiles of each *slimfast *paralog inspired the molecular evolutionary models we used to test if branches leading to male-biased paralogs experienced accelerated rates of evolution. Four codon-based branch models (depicted in Figure 3) [19,20] were constructed to test for differences in the ratio of non-synonymous to synonymous substitution rates along specific branches (*dN*/*dS *= ω). The null model (one ratio) assumed equal ω for all branches. The first alternative model (two ratios) assumed different ω ratios for branches within and outside the aphid-specific *slimfast *expansion. The second alternative model (three ratios) assumed an additional, collective ω ratio for the branches leading to the three male-biased paralogs, and the third alternative model (five ratios) assumed a distinct ω for each branch leading to a male-biased paralog or clade (Figure 3).

Results for the models and likelihood ratio tests are given in Table 3. The two ratios model had a significantly higher likelihood than the one ratio model and predicted a higher ω for branches within the aphid *slimfast *expansion, consistent with previous work [9]. Therefore, the remaining two models were compared to the two ratios model. The three ratios model was not significantly better than the two ratios model, but a significant improvement in likelihood was observed with the five ratios model. Using the five ratios model, branches leading to male-biased clades (*ACYPIslif6*, contig 1492 and *ACYPIslif5*, contig 8321) had lower ω values than the average branch within the expansion (0.17-0.18 vs. 0.22). In contrast, the branch leading to *ACYPIslif10 *had a greater ω value than other expansion branches (0.61 vs. 0.22).

**Table 3:** Molecular evolution results

Model	ω value	-lnL	**Null**^**a**^	P
One ratio	ω0 = 0.16415 ω1 = ω0 ω2 = ω0 ω3 = ω0 ω4 = ω0	26427.39		---
Two ratios	ω0 = 0.00452 ω1 = 0.22750 ω2 = ω1 ω3 = ω1 ω4 = ω1	26300.94	One ratio	**
Three ratios	ω0 = 0.00452 ω1 = 0.22624 ω2 = 0.23358 ω3 = ω2 ω4 = ω2	26300.92	Two ratios	NS
Five ratios	ω0 = 0.00451 ω1 = 0.22356 ω2 = 0.18305 ω3 = 0.17040 ω4 = 0.60752	26295.18	Two ratios	**

^a^Null model against which the given model was tested using a likelihood ratio test.

NS = Not Significant

** indicates that P < 0.05

## Discussion

The nutrient amino acid transporter *slimfast *plays an important role in nutritionally dependent processes, such as coordinated growth in both males and females and oogenesis in females. By acting as a nutrient sensor, *slimfast *stimulates processes like nutrient import, metabolism, and translation in response to nutrient availability via the Target of Rapamycin (TOR) signaling pathway [21,22]. TOR signaling activation relies especially on *slimfast *expression in the insect fat body, evidenced by the fact that in *Drosophila melanogaster*, downregulating *slimfast *in fat body cells disrupts overall growth [18]. In mosquitoes, *slimfast *expression in the fat body also activates vitellogenesis [23], a key process in oogenesis. While oogenesis is reproductive in nature, it is fundamentally nutrient-driven because female reproductive processes, unlike male reproductive processes, are energetically costly and depend directly on nutrient availability [24,25]. Although a male-specific role for *slimfast *has never been documented in any insect, we present evidence that some paralogs in the aphid-specific *slimfast *expansion have evolved a male functional role.

### Aphids possess male-enriched nutrient amino acid transporters, including conserved male-biased paralogs in the aphid-specific *slimfast *expansion

We have identified five nutrient amino acid transporters with at least 4-fold enriched expression in males relative to at least one female aphid morph. Although the software Blast2GO failed to annotate all five of the contigs we identified from the microarray (Table 1 and Additional file 3), InterProScan IDs and Pfam searches provided strong support that all five contigs are amino acid transporters and that four of the five contain either the *Aa_trans *or *AA_permease *transmembrane domains (Table 1). These domains characterize two amino acid transporter families, the amino acid/polyamine/organocation (APC) family (TC #2.A.3), and the amino acid/auxin permease (AAAP) family (TC #2.A.18) [26-29]. These amino acid transporter families contain all but a handful [30] of the known nutrient amino acid transporters. Though one contig (8321) did not significantly match any Pfam domain (Table 1), its strongly supported phylogenetic position within the *A. pisum *expansion of *slimfast *(Figure 1), a member of the APC family, indicates that contig 8321 is also a member of the APC family. Of the five male-enriched nutrient amino acid transporters (Table 1 and Additional file 3), contigs 1492 and 8321 (see Additional file 4 for GenBank accessions) are male-biased, being overexpressed in males relative to all three female reproductive morphs.

Shifting our study focus to *A. pisum *supported a conserved male role for *slimfast*. The phylogenetic placement of contigs 1492 and 8321 indicates that they are orthologous to *A. pisum slimfast *paralogs *ACYPIslif6 *and 5 respectively (Figure 1). Male-biased expression in these paralogs (Figure 2) suggests that their male-biased expression is conserved minimally in the Macrosiphini tribe, of which *A. pisum *and *M. persicae *are members. Further, QPCR expression analysis revealed one more male-biased paralog, *ACYPIslif10 *(Figure 2). Male expression in *ACYPIslif10 *was low compared to the expression observed for *ACYPIslif5 *and *6 *(Figure 2), which may indicate that it is expressed only in a particular tissue and its true expression level is confounded by quantifying expression in whole insects. Because *slimfast *is not known to have a male-specific role, the presence of male-biased *slimfast *paralogs in aphids is puzzling. If aphid *slimfast *paralogs retain a function in activating TOR signaling and nutrient-dependent processes, then the presence of male-biased paralogs could indicate that male and female aphids must overcome different nutritional challenges.

### The evolutionary origin of male-biased *slimfast *expression in aphids

Given the phylogenetic distribution of male-biased expression (Figure 2), we are unable to conclusively reconstruct when male-biased expression evolved within the *slimfast *expansion. The current data set can explain the distribution of male-biased expression by two alternatives. First, male-biased expression could be derived, which could have happened in one of two ways. Either male-biased expression evolved independently for each paralog, or it evolved twice independently and was lost in the lineage leading to *ACYPIslif4*. A second, equally parsimonious, explanation is that male-biased expression is the ancestral state and was lost three times. Derived and ancestral male-biased expression in the expansion are thus equally plausible given our data. Distinguishing between these two explanations would be facilitated by data for the three paralogs lacking expression information (Figure 2).

Despite the lack of resolution in our results for when male-biased expression evolved in the *slimfast *expansion, support for derived male-biased expression in aphids can be gleaned from the literature. In a study examining sex-biased genes in seven *Drosophila *species, *slimfast *was significantly female-biased in five species and not significantly enriched in either sex for the other two species (see Supplementary Tables from [31]). This expression may reflect the ancestral state of the aphid *slimfast *expansion since *Drosophila *genomes have only one *slimfast *copy (http://phylomedb.org), and the essential role of *slimfast *in growth [18] strongly suggests that *Drosophila slimfast *is under strong purifying selection. Our molecular evolution analyses support strong purifying selection for *Drosophila *and other insect *slimfast*, evidenced by the significantly lower ω found for branches outside the aphid *slimfast *expansion (Table 3). Further, the known female and sex-neutral roles [18,23] but lack of documented male role suggests that male-biased expression (and corresponding male function) is derived among aphids.

### Evolution of a male-biased functional role in aphid *slimfast*

Male-biased genes reflect traits that increase male fitness, such as male-male competition, sexually selected characters and secondary sexual characters [32]. The male roles of three aphid *slimfast *paralogs can be hypothesized from our current and previous results. Although we did not examine tissue-level expression in this study, our previous work [9] quantified relative expression levels of *slimfast *paralogs in asexual female head, gut and bacteriocytes. While tissue-level expression patterns may not be identical in both sexes, these data provide a framework within which to formulate some of the possible testable hypotheses about the function of male-biased paralogs. In this context, all three male-biased paralogs show highly enriched expression in asexual female gut [9], consistent with *slimfast *expression in *Drosophila *[18] and *Tribolium *[Supporting Table 3 from [33]].

The digestive tract interfaces between an animal and its diet, playing a critical role in uptaking dietary nutrients. Dietary nutrient availability is central to the aphid/*Buchnera *nutritional symbiosis because deficient dietary amino acids must be synthesized by *Buchnera*. While *Buchnera*-containing bacteriocytes are abundant in females, males contain relatively few [34] and in extreme cases, males completely lack bacteriocytes [35,36]. This sex-based difference in a major nutrient provisioning cell type suggests that males and females differ in their ability to synthesize amino acids deficient in their diet. Differential amino acid biosynthesis could be compensated for by upregulating amino acid transporters in the male gut, enabling greater uptake of certain amino acids from phloem sap.

In contrast, other expression patterns we observed previously suggest an alternative possibility for male function. In addition to being enriched in gut, two of the male-biased paralogs (*ACYPIslif6 *and *ACYPIslif10*; Figure 2) were also enriched in the asexual female head [9]. These paralogs could thus function in any of the head tissues included in the analysis, such as brain, eyes, mouthparts, antennae, or salivary glands. These tissues suggest that the male-biased paralogs could be implicated in sensory functions, such as locating a mate.

The possibility remains that the male-biased paralogs have evolved a different expression pattern (and function) from asexual females. One possible role for these male-biased paralogs that we cannot predict from female expression profiles is a role in male reproduction, such as spermatogenesis. Although *slimfast *has never been implicated in spermatogenesis, related mammalian transporters (*SLC7A1 *and *SLC7A2*) deliver L-Arginine to rat seminiferous tubule cells [37], where spermatogeneis begins. Thus, divergence of some *slimfast *paralogs to fill a male reproductive role is plausible. In light of the molecular evolution results, paralog *ACYPIslif10 *is the best candidate for having a role in spermatogenesis since its terminal branch has experienced an extremely accelerated rate of non-synonymous substitutions (Table 3). Accelerated evolutionary rates are commonly associated with male-biased genes, especially genes involved in sperm competition [32]. While the accelerated ω we observed is consistent with both positive and neutral/relaxed selection, both types of selection can lead to functional divergence [38,39]. Additional studies can tease apart the various hypotheses we have presented for the role played by male-biased *slimfast *paralogs in aphid biology.

### Insights on the maintenance of duplicate amino acid transporters in aphids

By pointing towards a derived evolutionary origin of male-biased function in *slimfast*, our results provide insights into a fundamental question of how the aphid genome retains the *slimfast *expansion and other nutrient amino acid transporter duplications [see also 9]. Given that most gene duplications fail to reach fixation [1], it is intriguing that the aphid genome possesses more nutrient amino acid transporters than other sequenced insects [9]. The presence of these duplicate amino acid transporters indicates that there must be a selective advantage to their maintenance. As mentioned above, we previously discovered that some duplicate nutrient amino acid transporters (including some *slimfast *paralogs) are highly enriched in bacteriocytes, leading us to hypothesize that these transporters mediate nutrient exchange across the *A. pisum*/*Buchnera *symbiotic interface [9]. A role in mediating endosymbiotic interactions strongly suggests that duplicate nutrient amino acid transporters and the *slimfast *expansion are maintained in the genome because they diverged to fill a novel role in symbiosis. The current study supports a different, but not mutually exclusive, hypothesis that the *slimfast *expansion (and possibly other nutrient amino acid transporter duplicates) is maintained because some paralogs diverged to fill novel, sex-specific roles. Future studies will be able to test the relative significance of symbiosis and sex in maintaining amino acid transporter gene duplications by examining the genetic architecture and expression of nutrient amino acid transporters in other phloem-feeding insects with different, less complex life cycles.

## Conclusions

This study is the first to report evidence for a male function of the nutrient amino acid transporter *slimfast*. Using a microarray and QPCR, we identified three male-biased *slimfast *paralogs in an aphid-specific gene family expansion, and two of those paralogs had conserved expression profiles across two aphid species. By integrating different sources of knowledge on the function and expression of *slimfast *and related nutrient amino acid transporters, we propose competing hypotheses for the function of male-biased *slimfast *in male-dependent nutritional, sensory, and reproductive functions.

This study highlights the necessity of examining different types of expression data for functionally annotating genes in novel genomes. While our previous work showed evidence that the aphid *slimfast *expansion is maintained in the *A. pisum *genome because some paralogs evolved a novel role in mediating symbiotic interactions [9], this study extends the hypothesis for the maintenance of the *slimfast *expansion to include paralog divergence for unexpected sex-dependent functions. The integration of different expression profiles to assist in functionally characterizing genes is particularly important for novel genes in all organisms, including gene duplications, which have the potential to evolve divergent functions from homologous genes present in single copies in other organisms.

## Methods

### Design, execution, and statistical analysis of microarray experiments

To investigate gene expression based on sex, Agilent microarrays targeting 10,478 *M. persicae *unigenes [40] and ESTs were used to quantify mRNA expression. The microarray measured gene expression between four treatments and two aphid lines (W109 and W115), both collected in 2008 on tobacco in Windsor, CT by ACCW and colleague. The four treatments were males and three different female reproductive morphs: sexual females, asexual females at short day, and asexual females at long day. We collected asexual females at long day from our standard asexual cultures (14:10 Light:Dark, 20°C). To induce sexual morphs, we simulated seasonal differences by changing the growth temperature and light/dark cycle (10:14 L:D, 16°C). When sexual morphs appeared, males, sexual females and asexual females at short day were collected.

Total RNA was extracted from ten flash frozen aphids per aphid line per treatment using an RNeasy Mini Kit (Qiagen, Valencia, CA). RNA quantity and quality was assessed using an RNA 6000 Chip on the Agilent 2100 Bioanalyzer. One microgram of total RNA was labeled using an Amino Allyl MessageAmp aRNA Amplification Kit (Ambion) and Cy3- and Cy5-NHS ethers (Amersham). Following labeling, the concentration of each sample was determined spectrophotometrically and equal amounts of labeled amplified RNA were competitively hybridized to the arrays. Factorial design was used for this microarray experiment. Four competitive hybridizations using the aphid line W109 (first biological replicate) were made as following: male → asexual female (long day) → sexual female → asexual female (short day) → male. The direction of the arrow indicates Cy3 → Cy5 sample labeling. An additional four competitive hybridizations were made with the reverse labeling (Cy5 → Cy3) using the aphid line W115 (second biological replicate).

Following hybridization and washing, microarrays were scanned at 5 μm resolution using a GenePix 4000B scanner (Molecular Devices). The resulting images were analyzed using GenePix Pro 6.1 (Molecular Devices) and subject to quality control using Acuity 4.0 (Molecular Devices). Data were analyzed using Linear Models for Microarray Data (LIMMA) implemented in R [41]. Data normalization was executed first within each array using loess normalization, then normalization between arrays was executed using the LIMMA quantile algorithm. Differential expression and false discovery rates (FDR) were assessed using a linear model and empirical Bayes moderated F statistics [42,43]. All primary microarray data are accessible through the National Center for Biotechnology Information (NCBI) Gene Expression Omnibus (GEO) database (http://www.ncbi.nih.gov/geo) under the GEO series accession number GSE31024.

### Annotating sex-enriched genes

Sex-enriched genes identified in the microarray were annotated in Blast2GO v. 12.7.0 [44]. Blast2GO implements a BLAST search and maps Gene Ontology (GO) annotations to the query sequences based on the best BLAST hits. Next, the annotation step evaluates candidate GO terms based on their hit similarity and annotation evidence codes (*e.g*. experimentally validated, computationally validated, etc.) to assign the most specific GO annotations possible to the original query sequences. The Blast2GO platform can also be used to perform an InterProScan to annotate sequences based on protein domain and motif signatures. GO terms corresponding to the InterPro annotation are then merged with the GO annotations assigned by the annotation step.

Sex-enriched *M. persicae *sequences were input as the query sequences and compared to the NCBI protein sequence database using a TBLASTN search, keeping up to 50 top hits at an e-value of 0.001 or less. Annotation was performed in two rounds to maximize both the quality and number of annotations. First, the annotation step was run with default evidence code weights, except that the evidence code IEA (Inferred from Electronic Annotation) was weighted 0. For the second round, unannotated sequences were passed through the annotation step using default evidence code weights. After the annotation step, we ran the InterProScan and merged the InterPro results with the previous annotations. Annotations were then augmented using Annex, a database of manually reviewed relationships between the three GO categories [45]. Finally, the GO-slim function was used to summarize the annotation results. A fisher exact test was implemented in Blast2GO to examine overrepresented GO terms in male-enriched and female-enriched genes. Significance was assessed by a FDR of 0.05 or less.

### Identification and annotation of male-enriched amino acid transporters

Male-enriched amino acid transporters were annotated based on GO terms and InterProScan IDs from a list of sex-biased genes found in the microarray. Annotations were manually validated by searching for protein domains in Pfam by hidden markov models (http://pfam.sanger.ac.uk/). Two male-biased amino acid transporters were assigned orthology to genes in the pea aphid, *A. pisum*, by reciprocal BLAST and phylogenetic analysis. Briefly, the *M. persicae *sequences were queried against the *A. pisum *non-redundant (nr) protein database on NCBI using a BLASTX search. Query sequences were contigs assembled from *M. persicae *ESTs [40] (available at http://www.aphidbase.com/aphidbase/downloads). The top *A. pisum *BLAST hit for each query was subsequently subjected to a local TBLASTN search against the entire set of *M. persicae *unigenes in the microarray.

For the phylogenetic analysis, male-biased *M. persicae *contigs were translated to protein and aligned to a profile of *A. pisum *homologs in SeaView v. 4 [46] using a Clustal W [47] plug-in. The profile, from Price *et al.*, [9], consisted of all *A. pisum *members of the *slimfast *gene family, *slimfast *sequences from select other insects, and two outgroup sequences closely related to the *slimfast *gene family [9]. The new alignment was then used to build a phylogeny in MrBayes v. 3.1.2 [48] using two simultaneous runs, each with four chains. Chains were run for two million generations, after which the standard deviation of split frequencies between the runs was less than 0.01. Convergence was assessed in Tracer v. 1.5 [49]. Briefly, Tracer plots the value of the log-likelihood and model parameters against the number of generations, visually displaying parameter convergence between the two runs and enabling us to determine that MrBayes sufficiently sampled the parameter space. A consensus tree was constructed after discarding generations making up the burn-in, as determined in Tracer v. 1.5 [49].

### Analyzing sex-biased gene expression of *slimfast *homologs in *A. pisum*

Sex-dependent expression for seven out of ten *A. pisum slimfast *homologs was quantified by performing QPCR on three different *A. pisum *clonal lineages that included two lineages with intermediate lifecycles, LSR1 [50] and 7A [51] and one holocyclic lineage, 9-2-1 [52] (see [53] for a description of the morphs produced by different aphid life cycle classes). The three paralogs for which we did not quantify expression shared so much sequence similarity and untranslated regions were so AT rich (~80%) that we were unable to design paralog-specific primers. For the two intermediate lineages (LSR1 and 7A), QPCR was performed on the same aphid morphs used in the microarray, asexual females at long day, asexual females at short day, sexual females, and males. For the holocyclic lineage (9-2-1), which completely switches to the production of sexual morphs at short day, QPCR was performed on asexual females at long day, sexual females, and males. Males and female morphs were collected as described above.

Total RNA was extracted from at least 6 whole, wingless adults of each morph using an RNeasy mini kit (Qiagen), and a DNase digest was performed to eliminate genomic DNA. cDNA was synthesized from 450 ng of total RNA using qScript cDNA SuperMix (Quanta Biosciences) and QPCR was performed using PerfeCTa^® ^SYBR^® ^Green FastMix^® ^(Quanta Biosciences). Gene expression was compared between morphs using 2^-ΔΔ*CT *^methodology [54], with expression between morphs normalized to the expression of housekeeping gene glyceraldehyde-3-dehydrogenase (*GAPDH*, ACYPI009769 [8]). Experiments were each performed in triplicate and included no template controls. Prior to running experiments, we verified that cDNA pools lacked genomic DNA by running no reverse transcription controls alongside no template controls and positive controls. QPCR reactions were performed on a Mastercycler^® ^ep realplex^4 ^real-time PCR system (Eppendorf) using a program that began with 95°C for 5 minutes, followed by 40 cycles at 95°C for 15 sec, 52°C for 15 sec, and 60°C for 20 sec. Amplification profiles and melt-curves were analyzed in Mastercycler^® ^ep realplex software v. 1.5 (Eppendorf). For information on primer sequences and efficiencies, see [9]. Gene expression data were collectively normalized, allowing comparison of expression profiles across paralogs and aphid lines. Normalization was performed by converting Δ*C*_T _values to z-scores as follows:

$$z=-10\times \left(\frac{\Delta C_{T}-\bar{\Delta C_{T}}}{\sigma_{\Delta C_{T}}}\right)$$

The normalized expression for each gene from the three lines was compiled into a heat map where yellow indicates z > 0 and blue indicates z < 0.

### Molecular evolution analyses

Rates of molecular evolution along phylogenetic tree branches were estimated for branches in the aphid specific *slimfast *expansion. The analysis was conducted using the same phylogeneny for which the methods were described above. The amino acid alignment used to reconstruct the tree was converted back to a codon alignment in SeaView v. 4 [46], and the codon alignment was used to estimate the ratio of non-synonymous (*dN*) to synonymous (*dS*) substitution rates along branches, or *dN*/*dS *(also denoted ω). The ω ratio reflects the strength and type of selection in protein coding sequences. Excess *dS *(ω < 1) indicates that selection favors substitutions that conserve the amino acid sequence (purifying selection), while equal *dS *and *dN *(ω = 1) illustrates relaxed selective constraints (neutral selection). Excess *dN *(ω > 1) indicates that selection favors substitutions that change the amino acid sequence (positive selection).

The ω ratio was estimated for different branches in the aphid *slimfast *expansion by maximum likelihood in the PAML package [55]. Analyses implemented the branch models [19,20] with one or more ω categories for branches and one ω across sites (model = 0 or 2, NSsites = 0). The parameters ω and κ (transition rate/transversion rate) were both estimated, starting from initial values of 0.2 and 2 respectively, and codon frequency was measured using a 3 × 4 codon table. We tested for elevated ω along specific branches using various models. Briefly, we tested the null model assuming equal ω across branches and several nested models allowing for different ω ratios for particular groups of branches and compared the log likelihoods under these models with a likelihood ratio test [LRT, [19]]. If the LRT showed a statistically significant improvement in log likelihood from one model to the next, then the model with the higher log likelihood was supported. Models we tested were inspired by the expression profiles of aphid paralogs in the expansion and we outline them in the results section.

## Authors' contributions

ACCW designed the microarray experiment. LN performed the image and statistical analysis on the microarray data. RPD identified and annotated sex-enriched genes from the microarray. ACCW and RPD conceived and designed the QPCR experiments. RPD performed the QPCR experiment. RPD conceived, designed and executed the molecular evolution analyses. RPD and ACCW wrote the paper. All authors have read and approved the final manuscript.

## Supplementary Material

Additional file 1 **Summary of BLAST, mapping, and annotation results from Blast2GO**. Table summarizing BLAST, mapping and annotation results from Blast2GO for sex-enriched genes identified in microarray. Table includes number of male-and female enriched genes and total number of genes that had significant BLAST hits (E < 0.001), mapped to GO terms, and were successfully annotated by Blast2GO. Click here for file

Additional file 2 **BLAST, mapping, and annotation results for female-enriched genes in microarray**. Table containing full BLAST, mapping and annotation results from Blast2GO for female-enriched genes identified in microarray. Includes data on fold-change, false discovery rate, and female morph comparisons. All female-enriched genes are listed, including those that were not successfully annotated in Blast2GO. See "Annotation" column for information on which genes were successfully annotated. GO terms are grouped by category: C = Cellular component, F = Molecular function, P = Biological process. Click here for file

Additional file 3 **BLAST, mapping, and annotation results for male-enriched genes in microarray**. Table containing full BLAST, mapping and annotation results from Blast2GO for male-enriched genes identified in microarray. Includes data on fold-change, false discovery rate, and female morph comparisons. All male-enriched genes are listed, including those that were not successfully annotated in Blast2GO. See "Annotation" column for information on which genes were successfully annotated. GO terms are grouped by category: C = Cellular component, F = Molecular function, P = Biological process. Click here for file

Additional file 4 ***Myzus persicae *unigenes (contigs) and Expressed Sequence Tags (ESTs) represented on microarray and corresponding GenBank accession numbers**. Left column contains *M. persicae *unigenes and ESTs. Unigenes (contigs) are numbered and ESTs have no contig ID (unassigned). Right column contains GenBank accession numbers corresponding to each contig and EST. Contig IDs listed multiple times are represented by multiple unique ESTs. Click here for file

Additional file 5 **Overrepresented Gene Ontology (GO) terms among sex-enriched contigs from microarray**. Results from fisher exact test for overrepresented GO terms among sex-enriched genes on the microarray. Includes GO ID, description, category (C = Cellular component, F = Molecular function, P = Biological process), sex in which the term was enriched, and details used to calculate the exact test. Click here for file

## References

[B1] Lynch M Conery J The evolutionary fate and consequences of duplicate genes Science 2000 290 1151 1155 10.1126/science.290.5494.1151 11073452

[B2] Colbourne JK Pfrender ME Gilbert D Thomas WK Tucker A Oakley TH Tokishita S Aerts A Arnold GJ Basu MK The Ecoresponsive Genome of Daphnia pulex Science 2011 331 555 561 10.1126/science.1197761 21292972 PMC3529199

[B3] IAGC Genome Sequence of the Pea Aphid Acyrthosiphon pisum Plos Biol 2010 8 e1000313 10.1371/journal.pbio.1000313 20186266 PMC2826372

[B4] Shigenobu S Bickel RD Brisson JA Butts T Chang C-C Christiaens O Davis GK Duncan EJ Ferrier DEK Iga M Comprehensive survey of developmental genes in the pea aphid, Acyrthosiphon pisum: frequent lineage-specific duplications and losses of developmental genes Insect Mol Biol 2010 19 47 62 20482639 10.1111/j.1365-2583.2009.00944.x

[B5] Jaubert-Possamai S Rispe C Tanguy S Gordon K Walsh T Edwards O Tagu D Expansion of the miRNA Pathway in the Hemipteran Insect Acyrthosiphon pisum Molecular Biology and Evolution 2010 27 979 987 10.1093/molbev/msp256 20179251 PMC2857804

[B6] Rider SD Srinivasan DG Hilgarth RS Chromatin-remodelling proteins of the pea aphid, Acyrthosiphon pisum (Harris) Insect Mol Biol 2010 19 201 214 20482651 10.1111/j.1365-2583.2009.00972.x PMC3845463

[B7] Huerta-Cepas J Marcet-Houben M Pignatelli M Moya A Gabaldon T The pea aphid phylome: a complete catalogue of evolutionary histories and arthropod orthology and paralogy relationships for Acyrthosiphon pisum genes Insect Mol Biol 2010 19 13 21 20482636 10.1111/j.1365-2583.2009.00947.x

[B8] Price DRG Tibbles K Shigenobu S Smertenko A Russell CW Douglas AE Fitches E Gatehouse AMR Gatehouse JA Sugar transporters of the major facilitator superfamily in aphids; from gene prediction to functional characterization Insect Mol Biol 2010 19 97 112 20482643 10.1111/j.1365-2583.2009.00918.x

[B9] Price DRG Duncan RD S S Wilson ACC Genome expansion and differential expression of amino acid transporters at the aphid/*Buchnera *symbiotic interface Mol Biol Evol 2011 10.1093/molbev/msr140 21613235

[B10] Miura T Braendle C Shingleton A Sisk G Kambhampati S Stern DL A comparison of parthenogenetic and sexual embryogenesis of the pea aphid Acyrthosiphon pisum (Hemiptera: Aphidoidea) J Exp Zoology B Mol Dev Evol: 2003 295 1 59 81 10.1002/jez.b.3 12548543

[B11] Srinivasan DG Fenton B Jaubert-Possamai S Jaouannet M Analysis of meiosis and cell cycle genes of the facultatively asexual pea aphid, Acyrthosiphon pisum (Hemiptera: Aphididae) Insect Mol Biol 2010 19 229 239 20482653 10.1111/j.1365-2583.2009.00960.x

[B12] Brisson JA Davis GK Stern DL Common genome-wide patterns of transcript accumulation underlying the wing polyphenism and polymorphism in the pea aphid (Acyrthosiphon pisum) Evol Dev 2007 9 338 346 10.1111/j.1525-142X.2007.00170.x 17651358

[B13] Karley AJ Ashford DA Mintob LM Pritchard J Douglas AE The significance of gut sucrase activity for osmoregulation in the pea aphid, *Acyrthosiphon pisum* Journal of Insect Physiology 2005 51 1313 1319 10.1016/j.jinsphys.2005.08.001 16169004

[B14] Price DRG Wilkinson HS Gatehouse JA Functional expression and characterisation of a gut facilitative glucose transporter, NIHT1, from the phloem-feeding insect Nilaparvata lugens (rice brown planthopper) Insect Biochemistry and Molecular Biology 2007 37 1138 1148 10.1016/j.ibmb.2007.07.001 17916500

[B15] Munson M Baumann P Kinsey M *Buchnera *gen. nov and *Buchnera aphidicola *sp. nov, a taxon consisting of the mycetocyte-associated, primary endosymbionts of aphids Int J Syst Bacteriol 1991 41 566 568 10.1099/00207713-41-4-566

[B16] Shigenobu S Wilson A Genomic revelations of a mutualism: the pea aphid and its obligate bacterial symbiont Cell Mol Life Sci 2011 10.1007/s00018-011-0645-2 PMC3064905 21390549

[B17] Zdobnov EM Apweiler R InterProScan - an integration platform for the signature-recognition methods in InterPro Bioinformatics 2001 17 847 848 10.1093/bioinformatics/17.9.847 11590104

[B18] Colombani J Raisin S Pantalacci S Radimerski T Montagne J Leopold P A nutrient sensor mechanism controls Drosophila growth Cell 2003 114 739 749 10.1016/S0092-8674(03)00713-X 14505573

[B19] Yang Z Likelihood ratio tests for detecting positive selection and application to primate lysozyme evolution Molecular Biology and Evolution 1998 15 568 573 9580986 10.1093/oxfordjournals.molbev.a025957

[B20] Bielawski J Yang Z Maximum likelihood methods for detecting adaptive evolution after gene duplication Journal of Structural and Functional Genomics 2003 12836699

[B21] Hundal HS Taylor PM Amino acid transceptors: gate keepers of nutrient exchange and regulators of nutrient signaling Am J Physiol-Endoc M 2009 296 E603 E613 10.1152/ajpendo.91002.2008 PMC2670634 19158318

[B22] Wullschleger S Loewith R Hall M TOR signaling in growth and metabolism Cell 2006 124 471 484 10.1016/j.cell.2006.01.016 16469695

[B23] Attardo GM Hansen IA Shiao S-H Raikhel AS Identification of two cationic amino acid transporters required for nutritional signaling during mosquito reproduction Journal of Experimental Biology 2006 209 3071 3078 10.1242/jeb.02349 16888056

[B24] Wheeler D The role of nourishment in oogenesis Annu Rev Entomol 1996 41 407 431 10.1146/annurev.en.41.010196.002203 15012335

[B25] Wigglesworth V Nutrition and reproduction in insects Proceedings of the Nutrition Society 1960 10.1079/pns19600007 13844694

[B26] Castagna M Shayakul C Trotti D Sacchi V Harvey W Hediger M Molecular characteristics of mammalian and insect amino acid transporters: Implications for amino acid homeostasis Journal of Experimental Biology 1997 200 269 286 9050235 10.1242/jeb.200.2.269

[B27] Saier M A functional-phylogenetic classification system for transmembrane solute transporters Microbiology and Molecular Biology Reviews 2000 64 354 411 10.1128/MMBR.64.2.354-411.2000 10839820 PMC98997

[B28] Saier M Tran C Barabote R TCDB: the Transporter Classification Database for membrane transport protein analyses and information Nucleic Acids Research 2006 34 D181 D186 10.1093/nar/gkj001 16381841 PMC1334385

[B29] Saier M Yen M Noto K Tamang D Elkan C The Transporter Classification Database: recent advances Nucleic Acids Research 2009 37 D274 D278 10.1093/nar/gkn862 19022853 PMC2686586

[B30] Boudko D Kohn A Meleshkevitch E Dasher M Seron T Stevens B Harvey W Ancestry and progeny of nutrient amino acid transporters Proc Natl Acad Sci USA 2005 102 1360 1365 10.1073/pnas.0405183101 15665107 PMC547818

[B31] Zhang Y Sturgill D Parisi M Kumar S Oliver B Constraint and turnover in sex-biased gene expression in the genus Drosophila Nature 2007 450 233 U232 10.1038/nature06323 17994089 PMC2386141

[B32] Ellegren H Parsch J The evolution of sex-biased genes and sex-biased gene expression Nat Rev Genet 2007 8 689 698 10.1038/nrg2167 17680007

[B33] Morris K Lorenzen M Hiromasa Y Tomich J Oppert C Elpidina E Vinokurov K Jurat-Fuentes J Fabrick J Oppert B Tribolium castaneum larval gut transcriptome and proteome: a resource for the study of the coleopteran gut Journal of Proteome Research 2009 8 3889 3898 10.1021/pr900168z 19545177

[B34] Douglas AE Mycetocyte Symbiosis in insects Biol Rev 1989 64 409 434 10.1111/j.1469-185X.1989.tb00682.x 2696562

[B35] Braendle C Miura T Bickel R Shingleton AW Kambhampati S Stern DL Developmental origin and evolution of bacteriocytes in the aphid-Buchnera symbiosis Plos Biol 2003 10.1371/journal.pbio.0000021 PMC212699 14551917

[B36] Fukatsu TIH Soldier and male of an eusocial aphid, *Colophina arma*, lack endosymbiont: Implications for physiological and evolutionary interactions between host and symbiont Journal of insect physiology 1992 38 1033 1042 10.1016/0022-1910(92)90012-3

[B37] Cerec V Piquet-Pellorce C Aly HAA Touzalin A-M Jegou B Bauche F Multiple pathways for cationic amino acid transport in rat seminiferous tubule cells Biol Reprod 2007 76 241 249 10.1095/biolreprod.106.056168 17065601

[B38] Lynch M Genome Expansion by Gene Duplication The origins of genome architecture 2007 Sunderland, Massechusetts: Sinauer Associates, Inc 193 236

[B39] Zhang J Evolution by gene duplication: an update Trends in Ecology & Evolution 2003 18 292 298 10.1016/S0169-5347(03)00033-821899825

[B40] Ramsey JS Wilson ACC de Vos M Sun Q Tamborindeguy C Winfield A Malloch G Smith DM Fenton B Gray SM Jander G Genomic resources for Myzus persicae: EST sequencing, SNP identification, and microarray design Bmc Genomics 2007 8 423 10.1186/1471-2164-8-423 18021414 PMC2213679

[B41] Smyth G Limma linear models for microarray data Bioinformatics and Computational Biology Solutions using R and Bioconductor 2005 New York: Springer 397 420

[B42] Smyth G Speed T Normalization of cDNA microarray data Methods 2003 31 265 273 10.1016/S1046-2023(03)00155-5 14597310

[B43] Smyth GK Linear models and empirical bayes methods for assessing differential expression in microarray experiments Stat Appl Genet Mol Biol 2004 3 Article3 16646809 10.2202/1544-6115.1027

[B44] Conesa A Gotz S Garcia-Gomez J Terol J Talon M Robles M Blast2GO: a universal tool for annotation, visualization and analysis in functional genomics research Bioinformatics 2005 21 3674 3676 10.1093/bioinformatics/bti610 16081474

[B45] Myhre S Tveit H Mollestad T Laegreid A Additional gene ontology structure for improved biological reasoning Bioinformatics 2006 22 2020 2027 10.1093/bioinformatics/btl334 16787968

[B46] Gouy M Guindon S Gascuel O SeaView Version 4: A Multiplatform Graphical User Interface for Sequence Alignment and Phylogenetic Tree Building Molecular Biology and Evolution 2010 27 221 224 10.1093/molbev/msp259 19854763

[B47] Thompson J Higgins D Gibson T Clustal-W - Improving the sensitivity of progressive multiple sequence alignment through sequence weighting, position-specific gap penalties and weight matrix choice Nucleic Acids Res 1994 22 4673 4680 10.1093/nar/22.22.4673 7984417 PMC308517

[B48] Huelsenbeck J Ronquist F MRBAYES: Bayesian inference of phylogenetic trees Bioinformatics 2001 17 754 755 10.1093/bioinformatics/17.8.754 11524383

[B49] Rambaut ADA Tracer v1.4 2007 http://beast.bio.ed.ac.uk/Tracer

[B50] Caillaud M Boutin M Braendle C Simon J A sex-linked locus controls wing polymorphism in males of the pea aphid, Acyrthosiphon pisum (Harris) Heredity 2002 89 346 352 10.1038/sj.hdy.6800146 12399992

[B51] Moran NA McLaughlin HJ Sorek R The Dynamics and Time Scale of Ongoing Genomic Erosion in Symbiotic Bacteria Science 2009 323 379 382 10.1126/science.1167140 19150844

[B52] Russell J Moran N Costs and benefits of symbiont infection in aphids: variation among symbionts and across temperatures P Roy Soc B-Biol Sci 2006 273 603 610 10.1098/rspb.2005.3348 PMC1560055 16537132

[B53] Wilson A Sunnucks P Hales D Heritable genetic variation and potential for adaptive evolution in asexual aphids (Aphidoidea) Biol J Linn Soc 2003 79 115 135 10.1046/j.1095-8312.2003.00176.x

[B54] Livak K Schmittgen T Analysis of relative gene expression data using real-time quantitative PCR and the 2(T)(-Delta Delta C) method Methods 2001 25 402 408 10.1006/meth.2001.1262 11846609

[B55] Yang Z PAML 4: Phylogenetic analysis by maximum likelihood Molecular Biology and Evolution 2007 24 1586 1591 10.1093/molbev/msm088 17483113

