# Supplementary material for: Novel male-biased expression in paralogs of the aphid slimfast nutrient amino acid transporter expansion
Source: BMC Evol Biol. 2011 Sep 14;11:253. doi: 10.1186/1471-2148-11-253 (PMC3231810; doi:10.1186/1471-2148-11-253)
Supplement: Additional file 1 — Summary of BLAST, mapping, and annotation results from Blast2GO. Table summarizing BLAST, mapping and annotation results from Blast2GO for sex-enriched genes identified in microarray. Table includes number of male-and female enriched genes and total number of genes that had significant BLAST hits (E < 0.001), mapped to GO terms, and were successfully annotated by Blast2GO. [file 1471-2148-11-253-S1.PDF]

**Additional file 1. Summary of BLAST, mapping, and annotation results from Blast2GO**

| | Number of genes | | |
|---|---|---|---|
| | Male | Female | Total |
| Total found in array | 768 | 725 | 1492* |
| Significant BLAST hits | 501 | 554 | 1054* |
| Mapping | | | |
| GO terms found | 371 | 414 | 784* |
| New annotations | | | |
| 1st Annotation | 202 | 291 | 492* |
| 2nd Annotation | 63 | 51 | 114 |
| InterProScan | 42 | 31 | 73 |
| Total annotated | 307 | 373 | 679* |

* One gene (Contig 2728) was enriched in males relative to asexual females and also enriched in sexual females relative to males
